# Targeting CBP and p300: Emerging Anticancer Agents

**DOI:** 10.3390/molecules29194524

**Published:** 2024-09-24

**Authors:** Domiziana Masci, Michela Puxeddu, Romano Silvestri, Giuseppe La Regina

**Affiliations:** 1 Department of Basic Biotechnological Sciences, Intensivological and Perioperative Clinics, Catholic University of the Sacred Heart, Largo Francesco Vito 1, 00168 Rome, Italy; domiziana.masci@unicatt.it; 2 Laboratory Affiliated to Istituto Pasteur Italia—Fondazione Cenci Bolognetti, Department of Drug Chemistry and Technologies, Sapienza University of Rome, Piazzale Aldo Moro 5, 00185 Rome, Italy; michela.puxeddu@uniroma1.it (M.P.); romano.silvestri@uniroma1.it (R.S.)

**Keywords:** cancer, CBP, p300, histone acetyltransferases, Wnt/β-catenin pathway, inhibitors, small molecules

## Abstract

CBP and p300 are versatile transcriptional co-activators that play essential roles in regulating a wide range of signaling pathways, including Wnt/β-catenin, p53, and HIF-1α. These co-activators influence various cellular processes such as proliferation, differentiation, apoptosis, and response to hypoxia, making them pivotal in normal physiology and disease progression. The Wnt/β-catenin signaling pathway, in particular, is crucial for cellular proliferation, differentiation, tissue homeostasis, and embryogenesis. Aberrant activation of this pathway is often associated with several types of cancer, such as colorectal tumor, prostate cancer, pancreatic and hepatocellular carcinomas. In recent years, significant efforts have been directed toward identifying and developing small molecules as novel anticancer agents capable of specifically inhibiting the interaction between β-catenin and the transcriptional co-activators CBP and p300, which are required for Wnt target gene expression and are consequently involved in the regulation of tumor cell proliferation, migration, and invasion. This review summarizes the most significant and original research articles published from 2010 to date, found by means of a PubMed search, highlighting recent advancements in developing both specific and non-specific inhibitors of CBP/β-catenin and p300/β-catenin interactions. For a more comprehensive view, we have also explored the therapeutic potential of CBP/p300 bromodomain and histone acetyltransferase inhibitors in disrupting the transcriptional activation of genes involved in various signaling pathways related to cancer progression. By focusing on these therapeutic strategies, this review aims to offer a detailed overview of recent approaches in cancer treatment that selectively target CBP and p300, with particular emphasis on their roles in Wnt/β-catenin-driven oncogenesis.

## 1. Introduction

CBP (CREB-binding protein) and its paralog p300 are pivotal transcriptional co-activators and histone acetyltransferases (HATs) that have been involved in several different biological functions including cell cycle regulation, proliferation, differentiation, apoptosis, and DNA damage response. Therefore, these proteins play critical roles in modulating multiple signaling pathways essential for maintaining cellular homeostasis and controlling gene expression. Due to their central regulatory functions, the overexpression or mutation of CBP and p300 has been strongly associated with various diseases, particularly cancer.

Among the numerous pathways modulated by CBP and p300, the p53 signaling pathway is one of the most extensively studied. As a key tumor suppressor, p53 governs crucial cellular processes such as cell cycle arrest, apoptosis, and DNA repair in response to cellular stress, often associated with the progression of neoplastic diseases. CBP and p300 acetylate p53 at the lysine residues K370, K372, K373, K381, K382, and K386, thereby stabilizing the protein and enhancing its transcriptional activity [1]. This acetylation enhances the DNA-binding ability of p53 and promotes its transcriptional activity, leading to growth arrest or apoptosis [2]. Therefore, since acetylation is crucial for p53’s antiproliferative and pro-apoptotic functions, the loss or mutation of its acetyltransferases in cancer underscores the importance of CBP and p300 in modulating this pathway, making their role particularly relevant for therapeutic intervention.

Another significant pathway regulated by CBP and p300 is the nuclear factor kappa-light-chain-enhancer of the activated B cell (NF-κB) signaling pathway, which controls the transcription of genes involved in immune responses, inflammation, apoptosis suppression and cell survival. Through the acetylation of key components of the NF-κB pathway, including the NF-κB proteins themselves (e.g., p65/RelA), CBP and p300 enhance the DNA-binding ability and transcriptional activity of NF-κB, leading to the upregulation of genes that promote cell survival, proliferation, and angiogenesis [3]. The NF-κB pathway has been reported to be especially relevant in inflammatory cancers, such as gastric and pancreatic cancers, where CBP/p300 inhibitors could potentially reduce NF-κB activity and suppress tumor-promoting inflammation.

In addition, CBP and p300 appear to be deeply involved in other critical signaling pathways, including the TGF-β/SMAD pathway, where they act as co-activators for SMAD transcription factors, which regulate gene expression involved in cell growth, differentiation, and immune responses [4]. The dysregulation of TGF-β signaling is implicated in cancer progression, and CBP/p300’s involvement in modulating SMAD3-dependent transcription suggests a therapeutic avenue for targeting cancers with altered TGF-β activity.

Moreover, CBP and p300 interact with the Notch signaling pathway, which is crucial for determining cell fate during differentiation. These co-activators promote the transcriptional activity of the Notch intracellular domain, which is relevant in cancers where Notch signaling is aberrantly activated, such as in certain leukemias and solid tumors [5]. Inhibition of CBP/p300 in these contexts may provide a means to disrupt this pathway and hinder tumor progression.

In the context of the hypoxia-inducible factor (HIF-1α) pathway, CBP and p300 facilitate the transcriptional activation of HIF-1α, a key regulator of the cellular response to hypoxia [6]. Hypoxic conditions in solid tumors lead to the upregulation of HIF-1α, which promotes tumor progression and metastasis. Therefore, the inhibition of the transcription factor-co-activator HIF-1α-p300/CBP interaction represents an attractive strategy to inhibit tumor growth under hypoxic conditions.

Finally, Wnt/β-catenin signaling pathway stands out as one of the most studied pathways modulated by CBP and p300. The binding of CBP to β-catenin preferentially activates genes associated with cellular self-renewal and proliferation, making it particularly relevant in the context of cancer stem cell biology. In contrast, the association of p300 with β-catenin is more closely linked to the initiation of genes involved in differentiation [7]. This distinction highlights the nuanced role that these co-activators play in cancer progression and emphasizes their importance as therapeutic targets. Aberrant activation of the Wnt/β-catenin pathway is well established in a range of cancers, including colorectal cancer, liver cancer, and leukemia, making CBP and p300 attractive targets for disrupting this signaling cascade.

Although CBP and p300 are known to be involved in several critical tumorigenic pathways, the first part of the present review will specifically focus on Wnt/β-catenin pathway, as detailed in Section 2, along with the inhibitors that target the interactions between the transcriptional co-activators CBP/p300 and β-catenin itself. Indeed, in Section 3, it will be explored how these interactions influence key cellular processes, such as proliferation and differentiation, and we will explore the latest advancements in the development of small-molecule inhibitors designed to disrupt CBP/p300 and β-catenin interactions, particularly in the context of cancer therapy.

Moreover, with the aim to provide a more comprehensive overview, we will also discuss CBP bromodomain and HAT inhibitors, which have garnered significant attention in recent years. While these inhibitors are not specific to Wnt/β-catenin signaling, they target the broader transcriptional roles of CBP/p300, affecting multiple signaling pathways involved in oncogenesis. Therefore, we believe that the inclusion of these inhibitors will enhance the overall scope of the review, offering a more complete perspective on the therapeutic potential of targeting CBP/p300.

## 2. Wnt/β-Catenin Signaling Pathway

The Wnt gene was originally identified from the integration-1 gene in mouse breast cancer and the wingless gene of Drosophila. Due to the similarities between these genes and their functional proteins, researchers combined the terms to form the name Wnt gene. Structurally, the Wnt/β-catenin pathway is comprised of four major segments: extracellular signal, membrane segment, cytoplasmatic segment and the nuclear one [8]. Extracellular signals are primarily mediated by Wnt proteins, including Wnt3a, Wnt1, and Wnt5a, which act as ligands to activate this pathway. The membrane segment mainly presents the Wnt receptors frizzled (Fzd), a specific sevenfold transmembrane receptor protein, and low-density lipoprotein receptor-related proteins (LRP) 5/6 (LRP 5/6). The cytoplasmic segment includes several proteins, such as glycogen synthase kinase-3β (GSK-3β), disheveled (DVL), casein kinase 1 (CK1), adenomatous polyposis coli (APC), and Axin, which facilitates the activation and translocation of β-catenin from the cytosol to nucleus, thereby triggers Wnt/β-catenin signaling pathway. The nuclear segment mainly involves β-catenin, T-cell factor (TCF)/lymphoid enhancer-binding factor (LEF) transcription factors, and β-catenin downstream target genes, such as matrix metalloproteinases (MMPs) and c-Myc [8].

The canonical Wnt pathway, also known as wingless/integrase-1 (Wnt)/β-catenin signaling pathway, plays a key role in several physiological processes, including cellular proliferation and differentiation, adult tissue homeostasis regeneration, cellular migration, and apoptosis [9,10,11,12,13]. Consequently, abnormal regulation of this pathway is often associated with different diseases, indicating its potential as a therapeutic target to treat several conditions [14]. In the cytosol, in the absence of an extracellular Wnt stimulus, β-catenin is first phosphorylated and subsequently ubiquitinated by the activity of a multiprotein destruction complex, which is composed of the Ser/Thr kinases glycogen synthase kinase 3 (GSK-3) and CK1, the scaffolding protein Axin, the APC protein, and the β-transducing repeats-containing proteins (β-TrCP). This process leads to β-catenin’s proteolytic degradation, thereby preventing β-catenin-mediated transcription [15].

**Figure 1:**
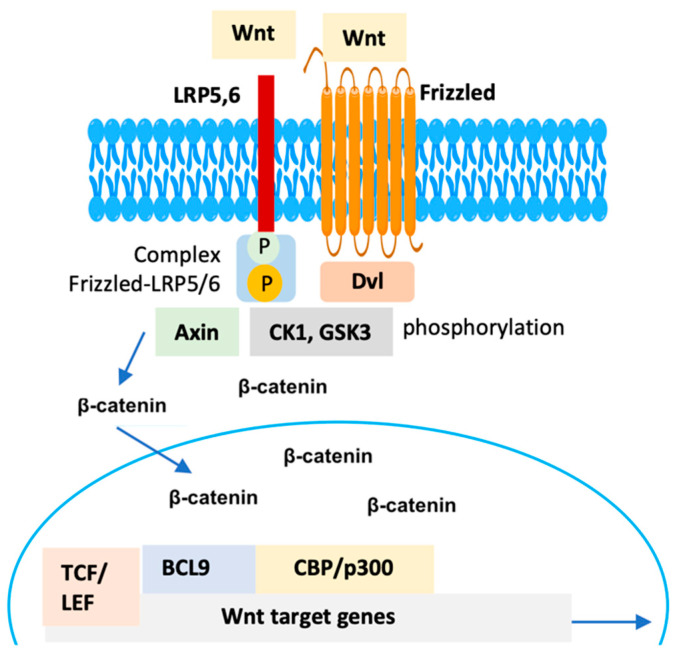
Overview of the canonical Wnt/β-catenin pathway upon activation by Wnt ligands.

Conversely, the Wnt/β-catenin pathway is activated when a Wnt ligand binds to the seven-pass transmembrane Fzd receptor and its co-receptor LRP6 or its close relative LRP5 (Figure 1). The binding of Wnt to Fzd and LRP5/6 forms a complex, which, along with the recruitment of the scaffolding protein DVL-1, leads to the phosphorylation and activation of LRP6. This activation subsequently recruits the Axin complex to the receptors, inhibiting the phosphorylation of β-catenin. As a result, β-catenin translocates and accumulates into the cell nucleus, where it activates the expression of T-cell factor (Tcf) and lymphoid enhancer-binding factor (LEF) transcription factors. Additionally, it recruits transcriptional co-activators such as B-cell lymphoma 9 (BCL9) [16,17] and its paralogue BCL9-like (BCL9L) [18], cAMP response element-binding protein (CREB)-binding protein (CBP)/p300 [19], and Pygopus (Pygo 1 or Pygo 2) [20]. Moreover, β-catenin induces epigenetic modifications [21] and, upon binding to E-cadherin, recruits actin filaments in the cytoplasm [22] (Figure 1).

Hence, it becomes apparent that the deregulation of Wnt/β-catenin signaling pathway strongly correlates with initiation and progression of various solid tumors and hematological malignancies [8,23,24,25]. Consequently, Wnt/β-catenin targeting agents have been developed and can be categorized into two major groups: small-molecule inhibitors [26] and monoclonal antibodies.

Small-molecule inhibitors, typically with a molecular weight of less than 500 Da, are generally predicted to have good oral absorption according to Lipinski’s rule of five [27]. Their *n*-octanol and water partition coefficient facilitates absorption through tissue and cell walls, and their volume of distribution indicates a propensity to redistribute the drug to other tissue compartments. Moreover, these small molecules can be precisely tailored to the receptor binding site through the hit-to-lead optimization process, which enhances their potency, selectivity, and pharmacokinetic properties. This optimization can even allow them to cross the blood–brain barrier, resulting in potential drug-like candidates with improved efficacy and safety profiles.

In contrast, monoclonal antibodies exhibit suboptimal oral bioavailability, limited tissue distribution, and lower concentrations in the target compartment. Additionally, they are more likely to induce immune responses. While small molecules bind to specific intra- or extracellular receptors with high affinity, monoclonal antibodies bind to extracellular and membrane-linked targets, activating the antitumor response of the body’s intrinsic immune system. Therefore, due to their ability to penetrate the cell nucleus and cross the blood–brain barrier, small-molecule drugs are a suitable choice for treating solid tumors; on the other hand, monoclonal antibodies, with their antigenic properties and large molecular weight, are better suited for the treatment of hematological tumors [28].

In the following section of the present review, we aim to provide a comprehensive analysis of recent advancements in targeting CBP and p300 with small-molecule inhibitors, highlighting their potential therapeutic applications in disrupting the Wnt/β-catenin signaling pathway. Through this analysis, we seek to elucidate the evidence supporting the critical role of p300 and CBP in oncogenesis and explore innovative strategies for cancer treatment.

## 3. CBP and p300

In order to generate a transcriptionally active complex, β-catenin recruits one of the following lysine acetyltransferase type 3 (KAT3) transcriptional co-activators called CBP (cyclic AMP response element-binding (CREB) binding protein) or its closely related homolog, p300, along with other components of the core transcriptional apparatus, thereby triggering the expression of a wide array of downstream target genes [29].

p300, also known as adenovirus early region 1 A (E1A)-associated 300 kDa protein, or KAT3B, is a member of the lysine acetyltransferases (KAT) superfamily and is involved in several biological functions, such as regulation of cell cycle, cell proliferation, differentiation and DNA repair [30,31]. p300 shows 63% homology at the amino-acid level with another member of the KAT superfamily called CBP. Both proteins are key epigenetic regulators that function through histone acetylation, a process that alters chromatin structure and regulates gene expression. Indeed, as histone acetyltransferases (HATs), they primarily acetylate lysine residue on histones H3 and H4, facilitating the release of DNA from nucleosomes. This chromatin relaxation allows greater access to the transcriptional machinery, enabling CBP and p300 to act as transcriptional co-factors and promote gene transcription. Through this mechanism, they play a pivotal role in regulating gene expression and influencing different cellular processes. Moreover, p300 and CBP play a crucial role in epigenetic regulation by acetylating several non-histone proteins, such as p53, p73, E2F, Myb, MyoD, HMG(I)Y, GATA1, and α-importin. This acetylation extends their influence beyond histone modification, linking various signaling pathways to changes in gene expression and modulating transcriptional activity.

Noteworthy, in addition to their role in transcription, p300 and CBP are also involved in DNA replication and repair, processes that require access to chromatin [29,30,31]. They interact with key proteins such as proliferating cell nuclear antigen (PCNA), flap endonuclease 1 (FEN1), DNA polymerase β, and thymine DNA glycosylase, with the latter three also serving as substrates for acetylation. These interactions further underscore the epigenetic influence of CBP and p300 in regulating essential cellular functions.

In other words, CBP and p300 play both common and distinct physiological roles, behaving as links between transcription cofactors of nuclear proteins, including oncoproteins and tumor-suppressor proteins [32,33,34], and the transcriptional machinery. Their involvement in chromatin remodeling and histone modifications is essential for transcriptional regulation, maintaining cellular identity, and regulating developmental processes. [35]. Moreover, they play a pivotal role in epigenetic processes, and their dysregulation has been implicated in the pathogenesis of various diseases. Indeed, it has been reported that CBP and p300 are crucial in human embryogenesis and their overexpression or mutation have been implicated in several diseases, especially leukemia/lymphoma, prostate cancer and other solid tumors [36]. 

In addition, their role in inflammatory and neurological disorders, as well as in cancer, through CBP/p300-dependent acetylation of H3K27, has also been well documented [37,38,39]. According to a recent review published by Robaszkiewicz [40], CBP/p300 inhibitors can be classified into three groups: catalytic competitors for the Lys-CoA binding pocket, inhibitors that interact with acetyl-lysine binding sites, and unrelated molecules such as NEO1132, NEO2734, and XP-524. 

In this section, we present a comprehensive overview of small-molecule inhibitors targeting CBP/β-catenin and p300/β-catenin emphasizing their potential applications in cancer treatment.

### 3.1. CBP/β-Catenin Inhibitors

The transcriptional co-activator proteins CBP and p300 are KAT3 protein acetyltransferases with high degree of homology. CBP and p300 are composed of multiple highly conserved domains, including a catalytic HAT domain, responsible for acetylating histones and other proteins, and an adjacent bromodomain that binds to acetylated histone tails. The conserved domains allow them to interact with several transcriptional regulators and other proteins [41,42]. Therefore, it is plausible to assume that these conserved domains represent promising targets for anticancer treatments.

**Figure 2:**
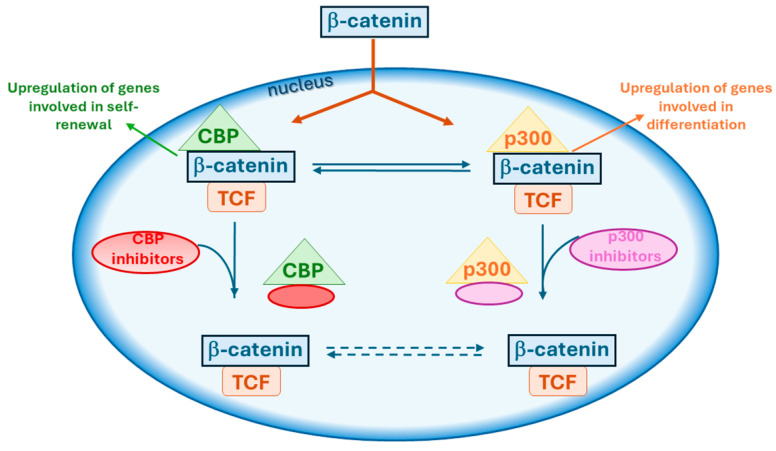
Model of inhibitors targeting β-catenin interaction with either CBP, promoting proliferation, or p300, driving differentiation.

The β-catenin signaling pathway shows a dichotomous behavior due to the interaction with these highly homologous co-activators, CBP and p300. Small-molecule modulators of these co-activators have demonstrated the ability to shift the balance between undifferentiated proliferation and differentiation (Figure 2), indicating their promising potential in treating diseases involving stem cells [43]. Selective antagonists of CBP/β-catenin have demonstrated to be effective in several preclinical tumor models, safely eliminating quiescent cancer stem cells (CSCs) without damaging multipotent somatic stem cells (SSCs) [44,45,46]. SSCs, such as hematopoietic and neural stem cells, can differentiate into various cell types within a specific tissue or organ. SSCs can also transform into CSCs, retaining the self-renewal capacity and differentiation potential of their normal SSC counterparts [47]. Several diseases have shown to benefit from treatment with CBP/β-catenin antagonists, for example, myocardial infarction [48], pulmonary fibrosis [49] and other types of fibrosis, as well as neuronal differentiation [50]. The observed benefits in preclinical models treated with CBP/β-catenin antagonists have been correlated with the activation of asymmetric differentiation of SSCs [51].

#### 3.1.1. Non-Specific Antagonists of CBP/β-Catenin Signaling

**Figure 3:**
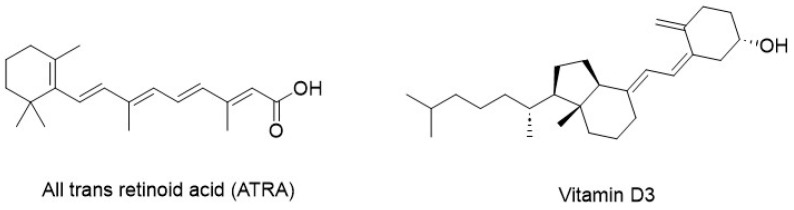
Non-specific antagonists of CBP/β-catenin signaling.

All-trans retinoic acid (ATRA, Figure 3) and arsenic trioxide (ATO) have been introduced as treatments for acute promyelocytic leukemia (APL), a subtype of acute myeloid leukemia characterized by the accumulation of promyelocytes, a type of immature white blood cell. Both ATRA and ATO, at 1 μM and 0.1–0.5 μM concentrations, respectively, act as differentiating agents and exhibit distinct behavior compared to agents currently used to treat various tumors [52]. The initial evidence of the differentiating properties of ATRA was observed in HL-60 cells, a model for APL, where ATRA promoted the differentiation of promyelocytes into fully mature granulocytes [53]. In healthy cells, the retinoic acid receptor alpha (RARα) binds to nuclear hormone receptor proteins known as retinoid X receptors (RXR), forming a heterodimeric structure [54]. This RARα-RXR heterodimer binds to retinoic acid response elements (RAREs) of DNA, which are involved in self-renewal and differentiation [52]. Upon binding of retinoic acid to RARα, conformational changes occur, enabling the release of co-repressors, recruitment of co-activators, chromatin remodeling, and subsequent gene expression [55]. 

Furthermore, ATRA has been shown to antagonize aberrant Wnt signaling in colorectal cancer (CRC) cells by binding of the RAR/RXR heterodimer to highly conserved sequences in the N-terminus of CBP, thus mimicking the CBP/β-catenin antagonism exerted by small molecules [56]. However, single treatment with ATRA alone has been demonstrated to be unable to induce durable remission of ALP. In contrast, the combination of ATRA with ATO has demonstrated robust synergistic activity, resulting in the expression of TGM2 and RARβ target genes and durable differentiation of the NB4 human APL cell line [57].

Vitamin D3 (Figure 3) is a micronutrient obtained from dietary sources or supplements and is also biosynthesized in the skin on exposure to solar ultraviolet B radiation. Low levels of vitamin D3 have been correlated with increased incidence of certain cancers [58]. The circulating form of vitamin D3, 25-hydroxyvitamin D [25(OH)D], represents the most reliable indicator of overall vitamin D3 status [59]. High levels of vitamin D3 have been associated with a reduced risk of colorectal and bladder cancers, whereas higher risk of prostate and possibly pancreatic cancers. However, surprisingly, in human studies, vitamin D3 have shown limited protective effects against cancer [58]. 

The vitamin D3 receptor (VDR), a nuclear receptor, forms a non-permissive heterodimeric complex with the retinoid X receptor (RXR). This RXR-VDR complex enhances VDR-mediated gene expression and vitamin D3-dependent transcription [60]. Vitamin D3 antagonizes the Wnt/β-catenin signaling pathway in CRC cells by binding of the RXR-VDR heterodimer to highly conserved sequence in the CBP N-terminus. Therefore, it has been demonstrated that, like ATRA, vitamin D3 acts as non-specific antagonist of CBP/β-catenin signaling. In fact, both ATRA and vitamin D3 induce the expression of genes that are present in the amino terminus of p300 via the L-SELL protein coding gene sequence [51].

#### 3.1.2. Specific Antagonists of CBP/β-Catenin Signaling 

**Figure 4:**
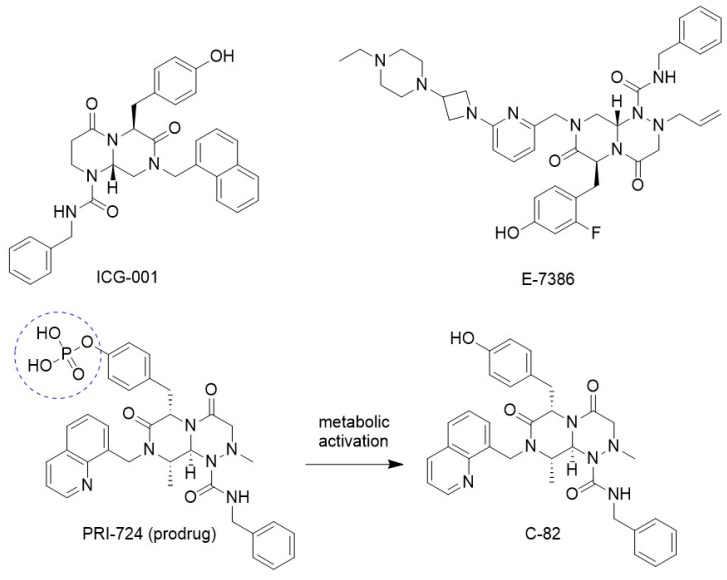
Specific antagonists of CBP/β-catenin signaling.

ICG-001 (Figure 4) was identified as an inhibitor of β-catenin-TCF transcriptional activity in SW480 colon carcinoma cells through screening a library of 5000 compounds using the TOP-Flash reporter assay, where it exhibited an IC_50_ value of 3 μM. Subsequent studies, using affinity chromatography, confirmed that ICG-001 specifically binds to the CBP co-activator, blocking its interaction with β-catenin without affecting β-catenin’s interaction with its closely related homolog p300 [61]. 

ICG-001 has been shown to inhibit the activation of pancreatic stellate cells, which are myofibroblast-like cells implicated in chronic pancreatitis and pancreatic cancer [62]. Additionally, ICG-001 inhibits the activation of hepatic stellate cells, which are functionally similar to pancreatic stellate cells [63,64]. By specifically antagonizing the CBP/β-catenin interaction, ICG-001 enhances the sensitivity of pancreatic cancer cells and tumors to the pyrimidine nucleoside analog gemcitabine [45]. IGC-001 also demonstrated its ability to inhibit primary tumor formation in Epstein–Barr virus (EBV)-associated metastatic nasopharyngeal carcinoma (NPC) through the miR-134/ITGB1 axis [65]. In human osteosarcoma cell lines KHOS, MG63, and 143B, ICG-001 inhibited cell proliferation by inducing a G0/G1 phase cell cycle blockade but also increased cell migration and metastatic dissemination to the lungs in osteosarcoma mouse models [66]. On the front of cancer stemness and metastasis, ICG-001 treatment reduced self-renewal activity and metastatic potential by suppressing MEIS1 expression [67]. Furthermore, ICG-001’s specific inhibition of CBP/β-catenin signaling promoted differentiation and sensitized quiescent, drug-resistant chronic myelogenous leukemia (CML) cells to Bcr-Abl tyrosine kinase inhibitors, such as imatinib [44].

PRI-724 (Figure 4), an analog of ICG-001, is a prodrug of the active metabolite C-82, which specifically inhibits the interaction between β-catenin and CBP. In lung fibroblasts cell nucleus, C-82 reduced CBP protein levels and increased the binding of β-catenin to p300. In lung fibroblasts treated with TGF-β, C-82 effectively inhibited the expression of α-smooth muscle actin, thereby preventing myofibroblast differentiation. In a mouse model of bleomycin-induced pulmonary fibrosis, a late administration of PRI-724 ameliorated pulmonary fibrosis. In addition, analysis of bronchoalveolar fluid (BALF) showed a decreased of the level of TGF-β1 in mice treated with PRI-724; while the production of TGF-β1 by alveolar macrophages was also inhibited by C-82. These results suggested PRI-724 is a potential antifibrotic agent [68,69]. Additionally, C-82 has been shown to inhibit proliferation of hepatocellular carcinoma cells with constitutively activated β-catenin, leading to an increased number of cells in the G0/G1 phase of the cell cycle and enhanced expression of apoptosis-related proteins [70].

E-7386 (Figure 4) has been reported as a selective inhibitor of the CBP/β-catenin interaction, exerting antitumor activity in tumor models with activated canonical Wnt signaling pathway [71]. Co-immunoprecipitation experiments in HEK293 cells overexpressing FLAG-tagged *N*-terminal region of CBP, treated with LiCl to activate the Wnt/β-catenin signaling pathway, revealed that E-7386 significantly reduces the CBP-β-catenin interaction. E-7386 also decreased the interaction between β-catenin and CBP in APC-mutated human gastric cancer ECC10 cells, known for their highly active Wnt/β-catenin signaling pathway. E-7386 inhibited TCF/LEF luciferase activity in HEK293 cells stably expressing TCF/LEF luciferase reporter and in ECC10 cells transfected with an expression vector for TCF/LEF luciferase reporter. 

In vivo, effectively inhibited tumor growth in the MMTV-Wnt1 isogenic mouse breast cancer model when administered at doses of 12.5–50 mg/kg orally twice daily for 7 days. Furthermore, E-7386 demonstrated synergistic antitumor effects when combined with an anti-PD-1 antibody in mouse preclinical models with activated Wnt/β-catenin signaling pathway [71]. Notably, in the Wnt1 tumor syngeneic mouse model, E-7386 alone exhibited significant antitumor activity, whereas the combination of E-7386 and anti-PD-1 mAb enhanced antitumor effects beyond those achieved by either treatment alone [72].

### 3.2. p300/β-Catenin Inhibitors

CBP and p300 play distinct roles of co-activators in the Wnt/β-catenin signaling cascade. CBP and p300 co-activators can induce stem cells towards proliferation/maintenance or differentiation state and are associated to several diseases in adult population [73,74]. Disrupting the CBP/β-catenin interaction activates differentiation in stem and progenitor cells (Figure 2), including human pluripotent stem cells [46] and cancer stem/progenitor cells [75]. 

To date, it is well established that the reduction in cellular p300 levels leads to a decrease in β-catenin/TCF transcription activity and inhibits β-catenin-mediated transformation. This underscores the critical role of p300 in β-catenin/TCF transcription and in the tumorigenesis that results from dysregulated β-catenin activity. Furthermore, it has been found that direct p300/β-catenin inhibition can maintain pluripotency of human pluripotent stem cells and mouse embryonic stem cells of Wnt-dependent signaling pathways [76].

#### 3.2.1. Specific Antagonists of p300/β-Catenin Signaling

**Figure 5:**
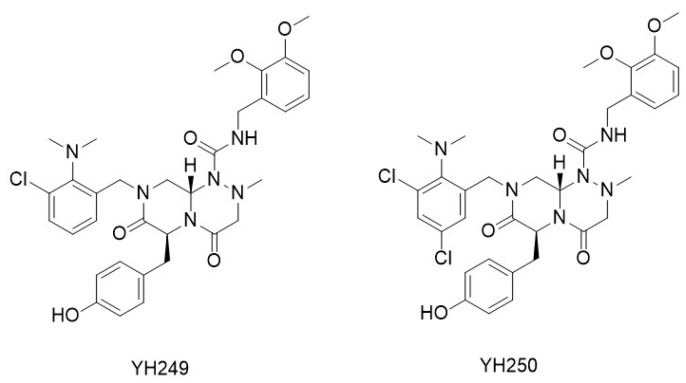
Specific antagonists of p300/β-catenin signaling.

YH249 and YH250 (Figure 5) were identified through the screening of a focused library of 90 compounds in HEK293 cells stably transfected with a SuperTopflash reporter. This assay, which evaluates both CBP and p300 co-activators, allowed for the selection of compounds with an IC_50_ < 10 μM for further testing in the survivin/luciferase reporter assay. This secondary assay aimed to identify selective p300/β-catenin antagonists, which were expected to inhibit SuperTopflash activity without affecting the survivin/luciferase reporter. Both YH249 and YH250 demonstrated over 200-fold selectivity for p300/β-catenin interactions compared to the homologous CBP/β-catenin interaction [76]. 

The direct binding interaction of YH240 to p300 was confirmed using a previously reported affinity column strategy [77] with a biotinylated derivative of YH249 (Bio-249) incorporating a propargyl amide variant. Both compounds showed to maintain the pluripotency of mouse and human embryonic stem cells, as well as human-induced pluripotent stem cells [65]. Additionally, YH250 was found to stimulate hematopoiesis in lethally or sub-lethally irradiated mice. Administration of a single dose of YH250 24 h post-irradiation significantly enhanced the proliferation of hematopoietic stem cells, prolonged survival, and improved the recovery of peripheral blood counts [78].

#### 3.2.2. Non-Specific Antagonists of p300/β-Catenin Signaling 

Similarly to the direct p300/β-catenin inhibition, indirect antagonism of p300/β-catenin has also been shown to maintain pluripotency in both human and mouse stem cells [76].

**Figure 6:**
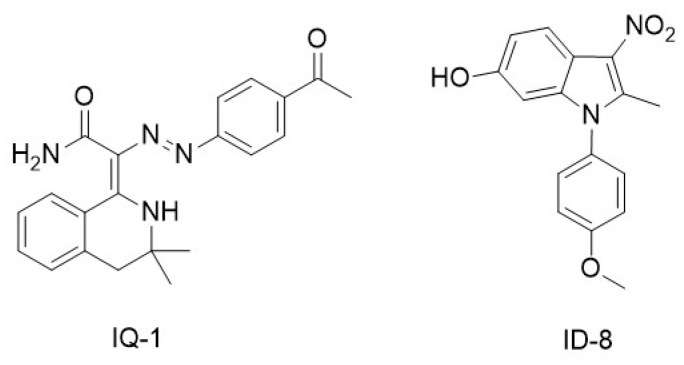
Non-specific antagonists of p300/β-catenin signaling.

IQ-1 (Figure 6) is a small molecule that was identified through a high-throughput cell-based assay. IQ-1 has been shown to maintain the pluripotency of murine embryonic stem cells in long-term culture by influencing Wnt-dependent signaling pathways. IQ-1 binds to the PR72/130 subunit of the heterotrimeric Nkd/PR72/PP2A complex, a serine/threonine phosphatase widely expressed in eukaryotic cells. Binding of IQ-1 to PR72/130 reduces the phosphorylation of p300 at Ser89, which concurrently increases the binding affinity of p300 to β-catenin. This results in a reduction in the interaction between p300 and β-catenin, while promoting the interaction between CBP and β-catenin. By preventing the switch from CBP to p300, IQ-1 facilitates the expansion of murine embryonic stem cells and retains their pluripotency without the need for murine embryonic fibroblasts or serum [79].

ID-8 (Figure 6) was originally screened along with IQ-1 in search for compounds able to maintain mouse embryonic stem cell pluripotency. ID-8 was shown to bind members of the DYRK kinase family [63], a target completely different from that of IQ-1. DYRKs (Dual-specificity tyrosine phosphorylation-regulated kinases) are conserved protein kinases present in yeast and humans. In humans, DYRKs can phosphorylate a broad set of proteins involved in many different cellular processes [80]. ID-8 enhances the CBP/β-catenin interaction in human embryonic stem cells, similarly to the action of IQ-1 in mouse embryonic stem cells, at the expense of the p300/β-catenin association. The different behavior of IQ-1 and ID-8 in human versus mouse embryonic stem cells was correlated to the different function/expression of PR72/130 and DYRKs in the two cell lines [46]. Furthermore, ID-8 enhanced human somatic cell reprogramming by upregulating pyruvate dehydrogenase kinase 4 (PDK4) and activating glycolysis [81]. 

In addition, ID-8 was identified as a potential compound that stimulates the proliferation of human kidney tubular epithelial cells following acute damage in vitro [82].

## 4. CBP/p300 Bromodomain Inhibitors

CBP and p300 possess HAT activity, which is crucial for chromatin modification. The reversible acetylation of histones modulates the compaction of genomic DNA within the cell nucleus. CBP specifically binds, through a conserved bromodomain, to the tumor suppressor protein p53, which is acetylated at lysine 382 in response to various cellular stress signals. This binding facilitates co-activator recruitment following DNA damage [83]. The crystal structure of the catalytic core of human p300, including its bromodomain, has been resolved at 2.8 Å [84]. Both CBP and p300 induce histone H3 lysine 27 (H3K27) acetylation, thereby activating gene transcription. Due to their high sequence homology and overlapping functions, CBP and p300 are often referred to as a single entity (CBP/p300) [38]. 

**Figure 7:**
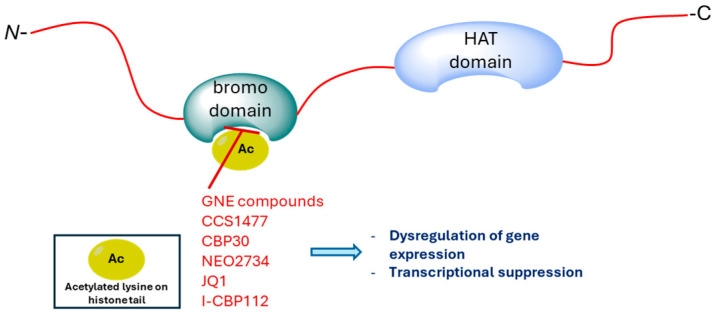
Proposed model of action of bromodomain inhibitors.

CBP/p300 are overexpressed in cancer cells, including those that are multidrug-resistant. They play a significant role in activating oncogene transcription, promoting cancer cell proliferation, survival, tumorigenesis, metastasis, immune evasion, and drug resistance [85]. In addition, inhibition of the CBP/p300 bromodomain is crucial for the viability of myeloma cells and represses the lymphocyte-specific transcription factor IRF4 target gene c-MYC [86]. Therefore, the CBP/p300 bromodomain inhibitors (Figure 7) act by interacting with acetyl-lysine binding sites, thereby blocking or limiting the interaction between the enzyme and chromatin.

In recent years, the development of CBP bromodomain inhibitors has garnered significant attention from research institutions and leading pharmaceutical companies due to their promising therapeutic potential in treating various cancers and other diseases. However, despite this interest, no CBP bromodomain inhibitors have been approved to date. Therefore, there is an urgent need to discover novel, potent, and specific CBP bromodomain inhibitors with diverse chemotypes to fully explore their therapeutic potential for various human cancers.

**Figure 8:**
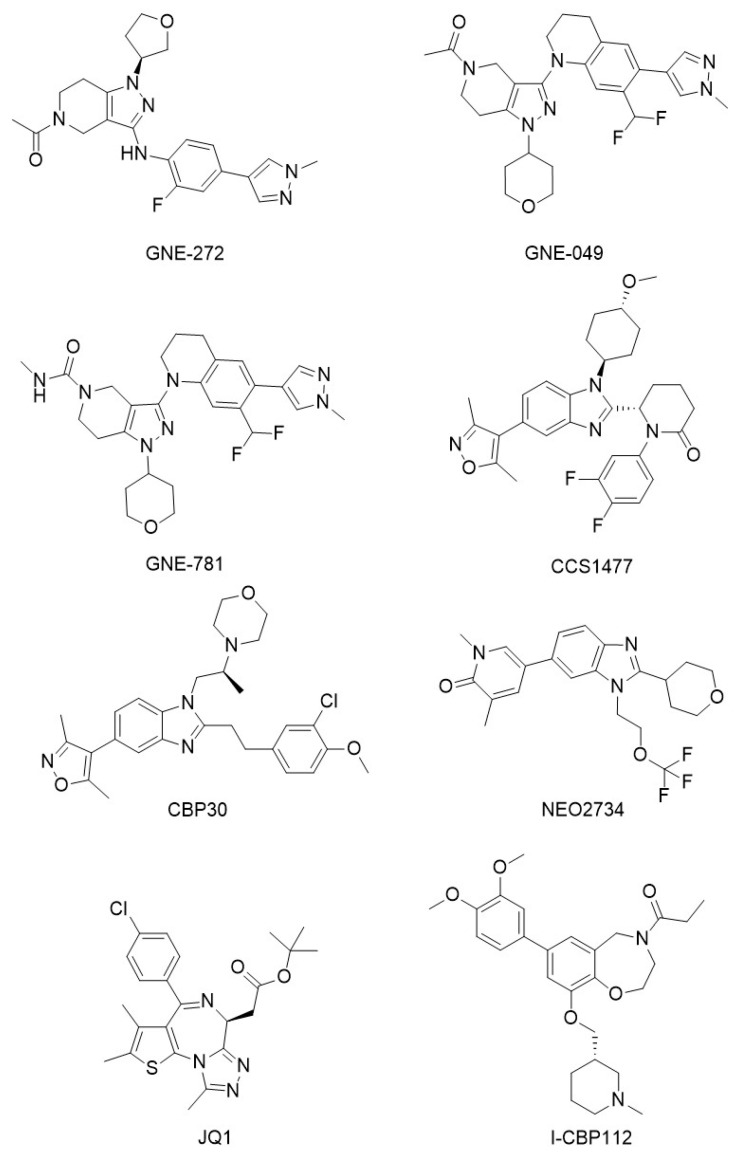
CBP/p300 bromodomain inhibitors.

GNE compounds. GNE-272 (Figure 8) is a CBP/p300 bromodomain inhibitor with 650-fold selectivity over the bromodomain 1 of the bromodomain-containing protein 4 [BRD4(1)] (BET bromodomain surrogate). GNE-272 was able to modulate CBP/p300 MYC expression and showed antitumor potential in MYC-dependent acute myeloid leukemia (AML) [87,88]. GNE-049 and GNE-781 (Figure 8) are constrained analogues of GNE-272. It has been demonstrated that GNE-049, a potent and selective inhibitor of CBP developed by Genentech, was able to block prostate cancer growth in vitro and in vivo. GNE-049 showed also potent inhibition of bromodomains of both CBP and p300, with IC_50_s of 1.1 and 2.3 nmol/L, respectively, and inhibited MYC expression with an EC_50_ of 14 nmol/L in MV-4-11 cells. GNE-049 did not impact androgen receptor (AR) levels but repressed the expression of AR target gene in a dose-dependent manner in AR expressing cell lines dependent on CBP/p300 for proliferation [89]. Similarly, GNE-781 exhibited highly potent and selective CBP/p300 inhibitory activity in a MOLM-16 AML xenograft model, demonstrating good in vivo pharmacokinetics, no central nervous system (CNS) penetration, and a dose-dependent decrease in Foxp3 transcript levels [90].

CCS1477 (Figure 8), developed by CellCentric, is an orally bioavailable and selective inhibitor small molecule of the CBP/p300 conserved common bromodomain that are critical transcriptional co-activators of the androgen receptor [91]. In addition, it represents the first CBP bromodomain inhibitor entering clinical trials. Targeting CBP/p300 via their conserved bromodomain, CCS1477 alleviates the oncogenic effects in castration-resistant prostate cancer (CRPC). CCS1477 was shown to bind CBP and p300 with Kd values of 1.7 and 1.3 nM, respectively, demonstrating only minimal binding to BRD2, 3, 4, 9 in a BROMOscan assay. CCS1477 exhibited potent growth inhibition of the AR-positive prostate cancer cell lines VCaP, 22Rv1 and LNCaP95 with IC_50_ values below 100 nM. In both 22Rv1 and LNCaP95 cell lines, CCS1477 reduced the expression of AR regulated genes and C-MYC protein after 48 h of treatment. CCS1477 significantly inhibited the growth of 22Rv1 mouse xenografts, with an associated reduction in AR signaling. In a patient-derived model of treatment-resistant lethal prostate cancer, CCS1477 inhibited tumor growth and androgen receptor signaling. In patients with advanced prostate cancer, CCS1477 modulated the levels of plasma kallikrein-related peptidase 3 (KLK3), a marker associated with prostate-specific antigen (PSA) [92] and regulated key prostate cancer therapeutic targets [91]. Furthermore, the combination of CCS1477 at its IC_50_ concentration with the nucleoside analog 5-azacytidine in MOLM-13 cells (a human leukemia cell line derived from an AML patient) showed a significant reduction in cell viability and synergistic activity. This synergy between CBP/p300 and 5-azacytidine enhances its cell-intrinsic response, providing significant insights for the treatment of hematological diseases [93].

CBP30 (Figure 8) is selective inhibitor of the CBP/p300 bromodomain that strongly reduces immune cell production of proinflammatory cytokines. In particular, CBP30 inhibited the secretion of cytokine IL-17A by Th17 cells in from healthy donors and from actively inflamed joints of patients with ankylosing spondylitis and psoriatic arthritis [94]. Spriano and co-workers [95] demonstrated that CBP30 showed >30-fold selectivity for CBP and p300 compared with other bromodomains, according to the human bromodomains screen performed by Hay et al. [93]. Of note, no binding to bromodomain testis-specific protein (BRDT) was detectable, and CBP30 exhibited only weak activity for the second bromodomains of bromo and extraterminal domain (BET) proteins. Treatment of K562 cells with CBP30 at 2 μM for 48 h induces rapid downregulation of MYC expression, suggesting that the antiproliferative effects in this cell line could be at least partially mediated by MYC, eventually with other transcription factors. Displacement of CBP and p300 from the hematopoietic GATA1 and MYC transcription factor binding sites results by CBP30 resulted in reduced histone acetylation at and consequent decrease in gene expression [96].

NEO2734 (Figure 8) is an orally active dual inhibitor selective for CBP/p300 and BET bromodomains, with IC_50_ in the nanomolar range. In a recent study conducted by Spriano and co-workers [95], the binding potency of NEO2734 was assessed against a panel of BRDs using a ligand-binding, site-directed competition assay. This studied demonstrated that NEO2734 binds both BET and CBP/EP300 proteins with nanomolar affinity. In particular, the Kds values for BRD2, BRD3, BRD4, and BRDT were in the single nanomolar range [97]. 

Additionally, exhibited antiproliferative activity across various cell lines, with particularly strong effects observed in those derived from leukemia, prostate cancer, and lymphomas. Diffuse large B-cell lymphoma (DLBCL) cells were particularly sensitive to NEO2734 as well. The compound demonstrated in vivo antitumor activity in activated B-cell (ABC)-DLBCL large B-cell lymphoma (TMD8) cell and AML leukemic MV-4-11 cell models. These findings were consistent with the results obtained in primary AML cells and patient-derived xenografts [98]. NEO2734 efficiently reduced the viability and induced apoptosis in primary AML cells, eliminated leukemic stem/progenitor cells from AML patient samples, and increased the efficacy of combination chemotherapy treatment in an in vivo AML patient-derived mouse model [99].

JQ1 (Figure 8) is a thienotriazolodiazepine compound that act as an inhibitor of the bromodomain-containing 4 proteins (BRD4). It exhibited anticancer activity by regulating BRD4-mediated transcriptional regulation. In addition, JQ1 has demonstrated anti-inflammatory and cardioprotective properties, further highlighting its potential therapeutic applications beyond oncology. It competitively binds to BRD4, inhibiting its transcriptional activation and suppressing the expression of BRD4 target genes. This action inhibits tumor cell proliferation and induces apoptosis in cancer cells [100]. JQ1 has demonstrated good therapeutic efficacy in several tumors, including the inhibition of testicular BRDT, which leads to testicular atrophy and reversible infertility, associated with the modulation of super-enriched gene expression. Furthermore, it has shown broad and potent inhibitory effects in various human AML cell lines and patient samples [101], further underscoring its potential as anticancer agent.

I-CBP112 (Figure 8) is a benzoxazepine compound identified through a research initiative aimed at developing CBP/p300 bromodomain inhibitors with superior affinity compared to JQ1 and I-BET76 BET inhibitors [102]. I-CBP112 showed potent and selective inhibition of CBP/p300, with minimal off-target effects on BET family bromodomains and on two G-protein coupled receptors (α1A and 5HT1). I-CBP112 acts as competitive inhibitor of protein-protein interactions involving acetyl-lysine. The (R)-isomer of I-CBP112 demonstrated to mimic the acetyl-lysine in a cocrystal complex with the bromodomain at 1.6 Å resolution. This interaction with the R1173 side chain formed a pocket accommodating the 3,4-dimethoxyphenyl ring. In addition, the combination of I-CBP112 and doxorubicin showed significant cytotoxicity in leukemia cells [91]. In breast, lung (A549) and hepatic (HepG2) cancer cell lines, I-CBP112 decreased the expression of some ATP-binding cassette (ABC) transporters associated with multidrug resistance in cancer cells. This reduction led to increased intracellular concentrations of chemotherapeutics such as doxorubicin, daunorubicin, and methotrexate. Specifically, in MDA-MB-231 cells, I-CBP112 repressed the gene promoters ABCC1 and ABCC10 and reduced trimethylation of histone H3 lysine 4 (H3K4me3), a modification associated with active transcription [103].

## 5. CBP/p300 HAT Inhibitors

CBP/p300 proteins are a subfamily of highly conserved HATs that act as acetylases on both histones, especially H3 and H4, and non-histone proteins, but also as co-activators or scaffold proteins in regulatory complexes [104,105]. CBP/p300 and p300/CBP-associated factor (PCAF) cooperate to acetylate the core histones in nucleosomes [39]. CBP/p300 HAT activity is required for CBP/p300-dependent gene transcription [106], while CBP/p300 bromodomain-dependent histone H3K27 acetylation drives the production of enhancer RNAs and its transcription [107]. CBP/p300 are also required to maintain cell identity. 

A recent work has also indicated that the inhibition of the CBP/EP300 bromodomain reduces the expression of genes specific to somatic cells, decreases acetylation of histone H3 on lysine 27 (H3K27Ac), and limits chromatin accessibility at targeted promoters and enhancers [108]. 

**Figure 9:**
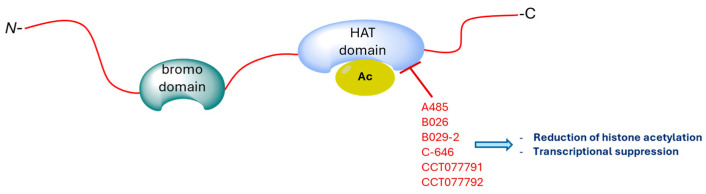
Proposed model of action of HAT inhibitors.

Reduction in CBP/p300 expression can lead to Rubinstein–Taybi syndrome, a genetic malformation syndrome associated with an increased propensity to develop tumors [109]. Additionally, this reduction significantly accelerated leukemogenesis in a mouse model of human myelodysplastic syndrome, primarily due to enhanced activation of the MAPK and JAK/STAT pathways, suppression of apoptosis, and the restoration of hematopoietic stem and progenitor cells [110]. Overexpression of CBP/p300 leads to increased transcription of oncogenes, promoting cancer cell proliferation, survival, tumorigenesis, metastasis, and immune evasion [85]. Furthermore, inhibition of CBP/p300 HAT activity induces global histone deacetylation and prevents the formation of pluripotent stem cell (Figure 9) [108].

**Figure 10:**
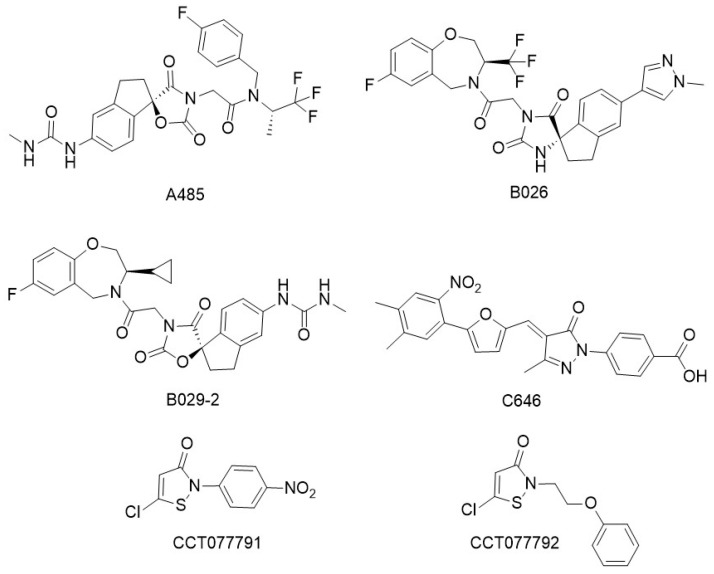
CBP/p300 HAT inhibitors.

A-485 (Figure 10) was reported as a potent, selective and drug-like catalytic inhibitor of CBP/p300 that competes with acetyl coenzyme A. A-485 selectively inhibits the proliferation of hematological malignancies and AR+ prostate cancer. Indeed, it has been shown to inhibit the androgen receptor transcriptional program in both androgen-sensitive and castration-resistant prostate cancer, effectively inhibiting tumor growth in a castration-resistant xenograft model [111]. Additionally, A-485 demonstrates selective inhibition of the nuclear protein in testis (NUT) midline carcinoma (NMC), a rare and highly aggressive form of undifferentiated squamous cell carcinoma typically occurring in the head, neck, and chest regions, characterized by chromosomal rearrangements involving the gene encoding NUT [112]. A-485 inhibits p300-mediated histone acetylation, resulting in the disruption of BRD4-NUT binding to hyperacetylated megadomains and the subsequent downregulation of the MYC, CCAT1 and TP63 associated genes [109].

B026 (Figure 10) is a selective and potent small-molecule HAT inhibitor, derived from the validated CBP/p300 inhibitor A485 [113], and developed through an artificial intelligence-assisted research project focused on identifying molecules containing structural elements conducive to inhibiting protein-protein interactions [114]. As a p300 HAT inhibitor, B026 was approximately 30-fold superior to A485, inhibiting cell growth in various hematological and androgen receptor positive (AR+) prostate cancers. This compound also exhibited a good pharmacokinetic profile and demonstrated efficacy in animal models of human cancer [106]. B026 inhibited the CBP with an IC_50_ value of 9.5 nM and was at least 2000-fold more selective over other HAT family members.

Following studies on B026, B029-2 (Figure 10) was developed [114] as another potent and selective small-molecule inhibitor of p300/CBP HAT, also derived from A-485 [111]. B029-2, which is structurally correlated to B026, showed superior inhibitory activity against p300, with an IC_50_ of 0.52 nmol/L, as detected by a radioactive acetyltransferase activity assay [115], and selectively inhibited the CBP with an IC_50_ of 11 nmol/L. Similar to A-485, B029-2 competes with Ac-CoA at the binding pocket. In hepatocellular carcinoma (HCC) xenografts, B029-2 efficiently inhibited the HAT activity of p300/CBP, leading to decrease the expression of cyclin D1 and epigenetic alteration of H3K27ac and H3K18ac.

C646 (Figure 10) is a highly selective small-molecule inhibitor of CBP, and p300 histone lysine acetyltransferases (KAT) discovered from a screening set of commercially available small molecules that were docked into the p300 HAT structure in the Lys-CoA binding pocket Compound C646 at 10 μM was highly selective against p300 with 86% inhibition and showed competitive mechanism versus acetyl-CoA with a Ki of 400 nM using steady-state kinetic analysis [116,117]. p300-HAT inhibitors could benefit patients with CBP-deficient cancers. C646 was able to suppress specifically the growth of CBP-deficient lung and hematopoietic cancer cells in vitro and in vivo [118]. C646 treatment demonstrated a significant reduction in cell growth, with 7/10 cell lines demonstrating > 50% reduction in growth in liquid culture [119]. C646 showed synergistic effect in combination with EZH2 in the treatment of solid tumors [120].

CCT077791 and CCT077792 (Figure 10) were identified by high-throughput screening of a compound library of 35 N-substituted isothiazolones as p300/(CREB binding protein) associated factor (PCAF), a member of the GCN5/PCAF family of nuclear histone acetyltransferases (HATs) [119], and p300 inhibitors. IC_50_ values were measured for the two peptide conjugates, Lys-CoA and H3- CoA-20, against the PCAF and p300 HAT enzymes. Both compounds inhibited cell proliferation and decreased global cellular acetylation in HCT116 and HT29 cancer cell lines. CCT077791 reduced total acetylation of histones H3 and H4, levels of specific acetylated lysine marks, and acetylation of alpha-tubulin [119]. The proposed mechanism of action was the irreversible binding with proteins detected by the sulforhodamine B assay [120] due to the reactivity of isothiazolones with cysteine residues. The thiol group of Cys177 probably reacts with isothiazolone ring to form a new covalent bond with consequent loss of HAT catalytic activity. According to the covalent bond hypothesis, HAT activity was not restored when PCAF was incubated with both CCT077791 and CCT077792, as reported in the study [111]. However, the exact mechanism of action remains unconfirmed, and an allosteric mechanism that also involves covalent bonding cannot be ruled out.

## 6. Conclusions

In this review, we examined small-molecule inhibitors, summarized in Table 1, targeting CBP/β-catenin, p300/β-catenin, CBP/p300 bromodomain, and CBP/p300 HAT, highlighting their potential applications in cancer treatment. Selective CBP/β-catenin antagonists proved to be effective and safe antitumor agents in several preclinical tumor models, with the ability to eliminate quiescent cancer stem cells. These CBP/β-catenin antagonists provide benefit in preclinical models, largely due to their ability to activate asymmetric differentiation of somatic stem cells. Specific antagonists of CBP/β-catenin signaling, while disrupting the CBP/β-catenin interaction, promote differentiation in stem and progenitor cells. 

Direct inhibition of p300/β-catenin has been shown to maintain the pluripotency of human pluripotent stem cells and mouse embryonic stem cells through Wnt-dependent signaling pathways. CBP/p300 are overexpressed in cancer cells, including the multidrug-resistant cancer cells. Inhibition of the CBP/P300 bromodomain has been found essential for repressing myeloma cell survival and lymphocyte-specific transcription factors. Additionally, inhibition of CBP/p300 histone acetyltransferase prevents the formation of pluripotent stem cells and induces the deacetylation of histones. 

CBP and p300 take part in various biological processes and cellular functions, including acting as transcriptional co-factors of oncoproteins, histone acetyltransferases, and influencing somatic mutations. Due to their involvement in numerous pathways, CBP and p300 are likely contributors to the oncogenesis of both hematological and solid tumors. Thus, CBP and p300 represent promising targets for developing novel agents to treat hematological and solid tumors.

## Figures and Tables

**Table 1:** Small-molecule inhibitors targeting CBP/β-catenin, p300/β-catenin, CBP/p300 bromodomain, and CBP/p300 HAT.

Category	Compounds	Ref.
Non-specific inhibitors of CBP/β-catenin signaling	ATRA, Vitamin D3	[51,56]
Specific inhibitors of CBP/β-catenin signaling	ICG-001, PRI-724, C-82, E-7386	[61,62,63,64,70,72]
Non-specific inhibitors of p300/β-catenin signaling	IQ-1, ID-8	[79,81]
Specific inhibitors of p300/β-catenin signaling	YH249, YH250	[76,77,78]
CBP/p300 bromodomain inhibitors	GNE-272, GNE0-49, GNE-781, CCS1477, CBP30, NEO2734, JQ1, I-CBP112	[87,88,89,91,94,95,100,102]
CBP/p300 HAT inhibitors	A-485, B026, B029-2, C646, CCT077791, CCT077792	[109,114,115,116,119]

## Data Availability

No new data were created in this study.

## Abbreviations

25(OH)D	25-hydroxyvitamin D
β-TrCP	β-transducing repeats-containing proteins
APC	adenomatous polyposis coli
APL	acute promyelocytic leukemia
ATO	arsenic trioxide
ATRA	all-trans retinoic acid
BCL9	B-cell lymphoma 9
BCL9L	B-cell lymphoma 9-like
[BRD4(1)]	bromodomain-containing protein 4
BRDT	bromodomain testis-specific protein
CBP	cyclic AMP response element-binding binding protein
CK1	casein kinase 1
CML	chronic myelogenous leukemia
CRC	colorectal cancer
CREB	cyclic AMP response element-binding
DLBCL	diffuse large B-cell lymphoma
DVL	disheveled
DVL-1	disheveled 1
DYRKs	dual-specificity tyrosine phosphorylation-regulated kinases
EBV	Epstein–Barr virus
Fzd	frizzled
GSK-3β	glycogen synthase kinase-3β
H3K27	histone H3 lysine 27
H3K27Ac	histone H3 lysine 27 acetylation
HAT	histone acetyltransferase
LEF	lymphoid enhancer-binding factor
LRP	low-density lipoprotein receptor-related proteins
LRP5	low-density lipoprotein receptor-related protein 5
LRP6	low-density lipoprotein receptor-related protein 6
MMPs	matrix metalloproteinases
NMC	NUT midline carcinoma
NPC	nasopharyngeal carcinoma
NUT	nuclear protein of the testis
RAREs	retinoic acid response elements
RARα	retinoic acid receptor alpha
RXR	retinoid X receptors
SSC	somatic stem cells
Tcf	T-cell factor

## References

[1] Grossman S.R. (2001). P300/CBP/P53 Interaction and Regulation of the P53 Response. Eur. J. Biochem..

[2] Nagasaka M., Miyajima C., Aoki H., Aoyama M., Morishita D., Inoue Y., Hayashi H. (2022). Insights into Regulators of P53 Acetylation. Cells.

[3] Zhou Y., Bastian I.N., Long M.D., Dow M., Li W., Liu T., Ngu R.K., Antonucci L., Huang J.Y., Phung Q.T. (2021). Activation of NF-ΚB and P300/CBP Potentiates Cancer Chemoimmunotherapy through Induction of MHC-I Antigen Presentation. Proc. Natl. Acad. Sci. USA.

[4] Wang Q., Xiong F., Wu G., Wang D., Liu W., Chen J., Qi Y., Wang B., Chen Y. (2023). SMAD Proteins in TGF-β Signalling Pathway in Cancer: Regulatory Mechanisms and Clinical Applications. Diagnostics.

[5] Dutta R., Tiu B., Sakamoto K.M. (2016). CBP/P300 Acetyltransferase Activity in Hematologic Malignancies. Mol. Genet. Metab..

[6] Albadari N., Deng S., Li W. (2019). The Transcriptional Factors HIF-1 and HIF-2 and Their Novel Inhibitors in Cancer Therapy. Expert. Opin. Drug Discov..

[7] Sun Y., Kolligs F.T., Hottiger M.O., Mosavin R., Fearon E.R., Nabel G.J. (2000). Regulation of β-Catenin Transformation by the P300 Transcriptional Coactivator. Proc. Natl. Acad. Sci. USA.

[8] Nusse R., Clevers H. (2017). Wnt/β-Catenin Signaling, Disease, and Emerging Therapeutic Modalities. Cell.

[9] Choi B.-R., Cave C., Na C.H., Sockanathan S. (2020). GDE2-Dependent Activation of Canonical Wnt Signaling in Neurons Regulates Oligodendrocyte Maturation. Cell Rep..

[10] Salik B., Yi H., Hassan N., Santiappillai N., Vick B., Connerty P., Duly A., Trahair T., Woo A.J., Beck D. (2020). Targeting RSPO3-LGR4 Signaling for Leukemia Stem Cell Eradication in Acute Myeloid Leukemia. Cancer Cell.

[11] Soleas J.P., D’Arcangelo E., Huang L., Karoubi G., Nostro M.C., McGuigan A.P., Waddell T.K. (2020). Assembly of Lung Progenitors into Developmentally-Inspired Geometry Drives Differentiation via Cellular Tension. Biomaterials.

[12] Valenta T., Hausmann G., Basler K. (2012). The Many Faces and Functions of β-Catenin. EMBO J..

[13] Clevers H., Nusse R. (2012). Wnt/β-Catenin Signaling and Disease. Cell.

[14] Coluccia A., Bufano M., La Regina G., Puxeddu M., Toto A., Paone A., Bouzidi A., Musto G., Badolati N., Orlando V. (2022). Anticancer Activity of (S)-5-Chloro-3-((3,5-Dimethylphenyl)Sulfonyl)-N-(1-Oxo-1-((Pyridin-4-Ylmethyl)Amino)Propan-2-Yl)-1H-Indole-2-Carboxamide (RS4690), a New Dishevelled 1 Inhibitor. Cancers.

[15] Rubinfeld B., Albert I., Porfiri E., Fiol C., Munemitsu S., Polakis P. (1996). Binding of GSK3β to the APC-β-Catenin Complex and Regulation of Complex Assembly. Science.

[16] Brack A.S., Murphy-Seiler F., Hanifi J., Deka J., Eyckerman S., Keller C., Aguet M., Rando T.A. (2009). BCL9 Is an Essential Component of Canonical Wnt Signaling That Mediates the Differentiation of Myogenic Progenitors during Muscle Regeneration. Dev. Biol..

[17] Cantù C., Zimmerli D., Hausmann G., Valenta T., Moor A., Aguet M., Basler K. (2014). Pax6-Dependent, but β-Catenin-Independent, Function of Bcl9 Proteins in Mouse Lens Development. Genes Dev..

[18] Kotolloshi R., Gajda M., Grimm M.-O., Steinbach D. (2022). Wnt/β-Catenin Signalling and Its Cofactor BCL9L Have an Oncogenic Effect in Bladder Cancer Cells. Int. J. Mol. Sci..

[19] Hecht A. (2000). The P300/CBP Acetyltransferases Function as Transcriptional Coactivators of Beta-Catenin in Vertebrates. EMBO J..

[20] Schwab K.R., Patterson L.T., Hartman H.A., Song N., Lang R.A., Lin X., Potter S.S. (2007). Pygo1 and Pygo2 Roles in Wnt Signaling in Mammalian Kidney Development. BMC Biol..

[21] Stewart D.J. (2014). Wnt Signaling Pathway in Non-Small Cell Lung Cancer. JNCI J. Natl. Cancer Inst..

[22] Huber A.H., Weis W.I. (2001). The Structure of the β-Catenin/E-Cadherin Complex and the Molecular Basis of Diverse Ligand Recognition by β-Catenin. Cell.

[23] Cao M.-Q., You A.-B., Zhu X.-D., Zhang W., Zhang Y.-Y., Zhang S.-Z., Zhang K., Cai H., Shi W.-K., Li X.-L. (2018). MiR-182-5p Promotes Hepatocellular Carcinoma Progression by Repressing FOXO3a. J. Hematol. Oncol..

[24] Zhang M., Weng W., Zhang Q., Wu Y., Ni S., Tan C., Xu M., Sun H., Liu C., Wei P. (2018). The LncRNA NEAT1 Activates Wnt/β-Catenin Signaling and Promotes Colorectal Cancer Progression via Interacting with DDX5. J. Hematol. Oncol..

[25] Spaan I., Raymakers R.A., van de Stolpe A., Peperzak V. (2018). Wnt Signaling in Multiple Myeloma: A Central Player in Disease with Therapeutic Potential. J. Hematol. Oncol..

[26] Nalli M., Masci D., Urbani A., La Regina G., Silvestri R. (2022). Emerging Direct Targeting β-Catenin Agents. Molecules.

[27] Lipinski C.A., Lombardo F., Dominy B.W., Feeney P.J. (2001). Experimental and Computational Approaches to Estimate Solubility and Permeability in Drug Discovery and Development Settings. Adv. Drug Deliv. Rev..

[28] Zhang Y., Wang X. (2020). Targeting the Wnt/β-Catenin Signaling Pathway in Cancer. J. Hematol. Oncol..

[29] Teo J.-L., Kahn M. (2010). The Wnt Signaling Pathway in Cellular Proliferation and Differentiation: A Tale of Two Coactivators. Adv. Drug Deliv. Rev..

[30] Giordano A., Avantaggiati M.L. (1999). P300 and CBP: Partners for Life and Death. J. Cell Physiol..

[31] Goodman R.H., Smolik S. (2000). CBP/P300 in Cell Growth, Transformation, and Development. Genes Dev..

[32] Eckner R., Ewen M.E., Newsome D., Gerdes M., DeCaprio J.A., Lawrence J.B., Livingston D.M. (1994). Molecular Cloning and Functional Analysis of the Adenovirus E1A-Associated 300-KD Protein (P300) Reveals a Protein with Properties of a Transcriptional Adaptor. Genes Dev..

[33] Avantaggiati M.L., Ogryzko V., Gardner K., Giordano A., Levine A.S., Kelly K. (1997). Recruitment of P300/CBP in P53-Dependent Signal Pathways. Cell.

[34] Marzio G., Wagener C., Gutierrez M.I., Cartwright P., Helin K., Giacca M. (2000). E2F Family Members Are Differentially Regulated by Reversible Acetylation. J. Biol. Chem..

[35] Chan H.M., La Thangue N.B. (2001). P300/CBP Proteins: HATs for Transcriptional Bridges and Scaffolds. J. Cell Sci..

[36] Iyer N.G., Özdag H., Caldas C. (2004). P300/CBP and Cancer. Oncogene.

[37] Durbin A.D., Wang T., Wimalasena V.K., Zimmerman M.W., Li D., Dharia N.V., Mariani L., Shendy N.A.M., Nance S., Patel A.G. (2022). EP300 Selectively Controls the Enhancer Landscape of MYCN -Amplified Neuroblastoma. Cancer Discov..

[38] Chen Q., Yang B., Liu X., Zhang X.D., Zhang L., Liu T. (2022). Histone Acetyltransferases CBP/P300 in Tumorigenesis and CBP/P300 Inhibitors as Promising Novel Anticancer Agents. Theranostics.

[39] Raisner R., Kharbanda S., Jin L., Jeng E., Chan E., Merchant M., Haverty P.M., Bainer R., Cheung T., Arnott D. (2018). Enhancer Activity Requires CBP/P300 Bromodomain-Dependent Histone H3K27 Acetylation. Cell Rep..

[40] Strachowska M., Robaszkiewicz A. (2024). Characteristics of Anticancer Activity of CBP/P300 Inhibitors—Features of Their Classes, Intracellular Targets and Future Perspectives of Their Application in Cancer Treatment. Pharmacol. Ther..

[41] Shi Y., Mello C. (1998). A CBP/P300 Homolog Specifies Multiple Differentiation Pathways in Caenorhabditis Elegans. Genes Dev..

[42] Dyson H.J., Wright P.E. (2005). Intrinsically Unstructured Proteins and Their Functions. Nat. Rev. Mol. Cell Biol..

[43] Ring A., Kim Y.-M., Kahn M. (2014). Wnt/Catenin Signaling in Adult Stem Cell Physiology and Disease. Stem Cell Rev. Rep..

[44] Zhao Y., Masiello D., McMillian M., Nguyen C., Wu Y., Melendez E., Smbatyan G., Kida A., He Y., Teo J.-L. (2016). CBP/Catenin Antagonist Safely Eliminates Drug-Resistant Leukemia-Initiating Cells. Oncogene.

[45] Manegold P., Lai K., Wu Y., Teo J.-L., Lenz H.-J., Genyk Y., Pandol S., Wu K., Lin D., Chen Y. (2018). Differentiation Therapy Targeting the β-Catenin/CBP Interaction in Pancreatic Cancer. Cancers.

[46] Gang E.J., Hsieh Y.-T., Pham J., Zhao Y., Nguyen C., Huantes S., Park E., Naing K., Klemm L., Swaminathan S. (2014). Small-Molecule Inhibition of CBP/Catenin Interactions Eliminates Drug-Resistant Clones in Acute Lymphoblastic Leukemia. Oncogene.

[47] Thomas P.D., Kahn M. (2016). Kat3 Coactivators in Somatic Stem Cells and Cancer Stem Cells: Biological Roles, Evolution, and Pharmacologic Manipulation. Cell Biol. Toxicol..

[48] Sasaki T., Hwang H., Nguyen C., Kloner R.A., Kahn M. (2013). The Small Molecule Wnt Signaling Modulator ICG-001 Improves Contractile Function in Chronically Infarcted Rat Myocardium. PLoS ONE.

[49] Henderson W.R., Chi E.Y., Ye X., Nguyen C., Tien Y., Zhou B., Borok Z., Knight D.A., Kahn M. (2010). Inhibition of Wnt/β-Catenin/CREB Binding Protein (CBP) Signaling Reverses Pulmonary Fibrosis. Proc. Natl. Acad. Sci. USA.

[50] Teo J.-L., Ma H., Nguyen C., Lam C., Kahn M. (2005). Specific Inhibition of CBP/β-Catenin Interaction Rescues Defects in Neuronal Differentiation Caused by a Presenilin-1 Mutation. Proc. Natl. Acad. Sci. USA.

[51] Kahn M. (2021). Taking the Road Less Traveled—The Therapeutic Potential of CBP/β-Catenin Antagonists. Expert. Opin. Ther. Targets.

[52] Jimenez J.J., Chale R.S., Abad A.C., Schally A.V. (2020). Acute Promyelocytic Leukemia (APL): A Review of the Literature. Oncotarget.

[53] Breitman T.R., Selonick S.E., Collins S.J. (1980). Induction of Differentiation of the Human Promyelocytic Leukemia Cell Line (HL-60) by Retinoic Acid. Proc. Natl. Acad. Sci. USA.

[54] Chambon P. (2005). The Nuclear Receptor Superfamily: A Personal Retrospect on the First Two Decades. Mol. Endocrinol..

[55] Kishimoto M., Fujiki R., Takezawa S., Sasaki Y., Nakamura T., Yamaoka K., Kitagawa H., Kato S. (2006). Nuclear Receptor Mediated Gene Regulation through Chromatin Remodeling and Histone Modifications. Endocr. J..

[56] Dillard A.C., Lane M.A. (2007). Retinol Decreases Β-catenin Protein Levels in Retinoic Acid-resistant Colon Cancer Cell Lines. Mol. Carcinog..

[57] Huynh T.T., Sultan M., Vidovic D., Dean C.A., Cruickshank B.M., Lee K., Loung C.-Y., Holloway R.W., Hoskin D.W., Waisman D.M. (2019). Retinoic Acid and Arsenic Trioxide Induce Lasting Differentiation and Demethylation of Target Genes in APL Cells. Sci. Rep..

[58] Mondul A.M., Weinstein S.J., Layne T.M., Albanes D. (2017). Vitamin D and Cancer Risk and Mortality: State of the Science, Gaps, and Challenges. Epidemiol. Rev..

[59] Giovannucci E. (2005). The Epidemiology of Vitamin D and Cancer Incidence and Mortality: A Review (United States). Cancer Causes Control.

[60] Bettoun D.J., Burris T.P., Houck K.A., Buck D.W., Stayrook K.R., Khalifa B., Lu J., Chin W.W., Nagpal S. (2003). Retinoid X Receptor Is a Nonsilent Major Contributor to Vitamin D Receptor-Mediated Transcriptional Activation. Mol. Endocrinol..

[61] Kahn M. (2014). Can We Safely Target the WNT Pathway?. Nat. Rev. Drug Discov..

[62] Omary M.B., Lugea A., Lowe A.W., Pandol S.J. (2007). The Pancreatic Stellate Cell: A Star on the Rise in Pancreatic Diseases. J. Clin. Investig..

[63] Che M., Kweon S.-M., Teo J.-L., Yuan Y.-C., Melstrom L.G., Waldron R.T., Lugea A., Urrutia R.A., Pandol S.J., Lai K.K.Y. (2020). Targeting the CBP/β-Catenin Interaction to Suppress Activation of Cancer-Promoting Pancreatic Stellate Cells. Cancers.

[64] Buchholz M., Kestler H.A., Holzmann K., Ellenrieder V., Schneiderhan W., Siech M., Adler G., Bachem M.G., Gress T.M. (2005). Transcriptome Analysis of Human Hepatic and Pancreatic Stellate Cells: Organ-Specific Variations of a Common Transcriptional Phenotype. J. Mol. Med..

[65] Chen L., Chiang Y.C., Chan L.S., Chau W.Y., Lung M.L., Kahn M., Lo K.W., Mak N.K., Lung H.L. (2022). The CBP/β-Catenin Antagonist, ICG-001, Inhibits Tumor Metastasis via Blocking of the MiR-134/ITGB1 Axis-Mediated Cell Adhesion in Nasopharyngeal Carcinoma. Cancers.

[66] Danieau G., Morice S., Renault S., Brion R., Biteau K., Amiaud J., Cadé M., Heymann D., Lézot F., Verrecchia F. (2021). ICG-001, an Inhibitor of the β-Catenin and CAMP Response Element-Binding Protein Dependent Gene Transcription, Decreases Proliferation but Enhances Migration of Osteosarcoma Cells. Pharmaceuticals.

[67] Choi J.-H., Jang T.-Y., Jeon S.-E., Kim J.-H., Lee C.-J., Yun H.-J., Jung J.-Y., Park S.-Y., Nam J.-S. (2021). The Small-Molecule Wnt Inhibitor ICG-001 Efficiently Inhibits Colorectal Cancer Stemness and Metastasis by Suppressing MEIS1 Expression. Int. J. Mol. Sci..

[68] Okazaki H., Sato S., Koyama K., Morizumi S., Abe S., Azuma M., Chen Y., Goto H., Aono Y., Ogawa H. (2019). The Novel Inhibitor PRI-724 for Wnt/β-Catenin/CBP Signaling Ameliorates Bleomycin-Induced Pulmonary Fibrosis in Mice. Exp. Lung Res..

[69] Ding H., Chen J., Qin J., Chen R., Yi Z. (2021). TGF-β-Induced α-SMA Expression Is Mediated by C/EBPβ Acetylation in Human Alveolar Epithelial Cells. Mol. Med..

[70] Gabata R., Harada K., Mizutani Y., Ouchi H., Yoshimura K., Sato Y., Kitao A., Kimura K., Kouji H., Miyashita T. (2020). Anti-Tumor Activity of the Small Molecule Inhibitor PRI-724 Against β-Catenin-Activated Hepatocellular Carcinoma. Anticancer Res..

[71] Yamada K., Hori Y., Inoue S., Yamamoto Y., Iso K., Kamiyama H., Yamaguchi A., Kimura T., Uesugi M., Ito J. (2021). E7386, a Selective Inhibitor of the Interaction between β-Catenin and CBP, Exerts Antitumor Activity in Tumor Models with Activated Canonical Wnt Signaling. Cancer Res..

[72] Hori Y., Yamada K., Kato Y., Ozawa Y., Odagami T., Matsui J., Matsushima T., Nomoto K., Kouji H., Owa T. (2017). Abstract 5172: E7386, an Orally Active CBP/Beta-Catenin Modulator, Induces T Cells Infiltration into Tumor and Enhances Antitumor Activity of Anti-PD-1 MAb in Wnt1 Tumor Syngeneic Mice Model. Cancer Res..

[73] Kahn M. (2011). Symmetric Division Versus Asymmetric Division: A Tale of Two Coactivators. Future Med. Chem..

[74] Hasegawa K., Yasuda S., Teo J.-L., Nguyen C., McMillan M., Hsieh C.-L., Suemori H., Nakatsuji N., Yamamoto M., Miyabayashi T. (2012). Wnt Signaling Orchestration with a Small Molecule DYRK Inhibitor Provides Long-Term Xeno-Free Human Pluripotent Cell Expansion. Stem Cells Transl. Med..

[75] Higuchi Y., Nguyen C., Yasuda S.-Y., McMillan M., Hasegawa K., Kahn M. (2016). Specific Direct Small Molecule P300/β-Catenin Antagonists Maintain Stem Cell Potency. Curr. Mol. Pharmacol..

[76] Zhao Y., Wu K., Nguyen C., Smbatyan G., Melendez E., Higuchi Y., Chen Y., Kahn M. (2017). Small Molecule P300/Catenin Antagonist Enhances Hematopoietic Recovery after Radiation. PLoS ONE.

[77] Emami K.H., Nguyen C., Ma H., Kim D.H., Jeong K.W., Eguchi M., Moon R.T., Teo J.-L., Oh S.W., Kim H.Y. (2004). A Small Molecule Inhibitor of β-Catenin/Cyclic AMP Response Element-Binding Protein Transcription. Proc. Natl. Acad. Sci. USA.

[78] Miyabayashi T., Teo J.-L., Yamamoto M., McMillan M., Nguyen C., Kahn M. (2007). Wnt/β-Catenin/CBP Signaling Maintains Long-Term Murine Embryonic Stem Cell Pluripotency. Proc. Natl. Acad. Sci. USA.

[79] Boni J., Rubio-Perez C., López-Bigas N., Fillat C., de la Luna S. (2020). The DYRK Family of Kinases in Cancer: Molecular Functions and Therapeutic Opportunities. Cancers.

[80] Xu J., Fang S., Wang N., Li B., Huang Y., Fan Q., Shi J., Liu H., Shao Z. (2022). Dual-Specificity Tyrosine Phosphorylation-Regulated Kinase Inhibitor ID-8 Promotes Human Somatic Cell Reprogramming by Activating PDK4 Expression. Stem Cell Rev. Rep..

[81] Monteiro M.B., Ramm S., Chandrasekaran V., Boswell S.A., Weber E.J., Lidberg K.A., Kelly E.J., Vaidya V.S. (2018). A High-Throughput Screen Identifies DYRK1A Inhibitor ID-8 That Stimulates Human Kidney Tubular Epithelial Cell Proliferation. J. Am. Soc. Nephrol..

[82] Mujtaba S., He Y., Zeng L., Yan S., Plotnikova O., Sachchidanand, Sanchez R., Zeleznik-Le N.J., Ronai Z., Zhou M.-M. (2004). Structural Mechanism of the Bromodomain of the Coactivator CBP in P53 Transcriptional Activation. Mol. Cell.

[83] Delvecchio M., Gaucher J., Aguilar-Gurrieri C., Ortega E., Panne D. (2013). Structure of the P300 Catalytic Core and Implications for Chromatin Targeting and HAT Regulation. Nat. Struct. Mol. Biol..

[84] Jin Q., Yu L.-R., Wang L., Zhang Z., Kasper L.H., Lee J.-E., Wang C., Brindle P.K., Dent S.Y.R., Ge K. (2011). Distinct Roles of GCN5/PCAF-Mediated H3K9ac and CBP/P300-Mediated H3K18/27ac in Nuclear Receptor Transactivation. EMBO J..

[85] Conery A.R., Centore R.C., Neiss A., Keller P.J., Joshi S., Spillane K.L., Sandy P., Hatton C., Pardo E., Zawadzke L. (2016). Bromodomain Inhibition of the Transcriptional Coactivators CBP/EP300 as a Therapeutic Strategy to Target the IRF4 Network in Multiple Myeloma. Elife.

[86] Romero F.A., Magnuson S., Pastor R., Tsui V.H.-W., Murray J., Crawford, Albrecht B.K., Cote A., Taylor A.M., Lai K.W. (2016). 4,5,6,7-Tetrahydro-1H-Pyrazolo [4,3-C]Pyridin-3-Amine Compounds as CBP and/or EP300 Inhibitors.

[87] Jin L., Garcia J., Chan E., de la Cruz C., Segal E., Merchant M., Kharbanda S., Raisner R., Haverty P.M., Modrusan Z. (2017). Therapeutic Targeting of the CBP/P300 Bromodomain Blocks the Growth of Castration-Resistant Prostate Cancer. Cancer Res..

[88] Romero F.A., Murray J., Lai K.W., Tsui V., Albrecht B.K., An L., Beresini M.H., de Leon Boenig G., Bronner S.M., Chan E.W. (2017). GNE-781, A Highly Advanced Potent and Selective Bromodomain Inhibitor of Cyclic Adenosine Monophosphate Response Element Binding Protein, Binding Protein (CBP). J. Med. Chem..

[89] Welti J., Sharp A., Brooks N., Yuan W., McNair C., Chand S.N., Pal A., Figueiredo I., Riisnaes R., Gurel B. (2021). Targeting the P300/CBP Axis in Lethal Prostate Cancer. Cancer Discov..

[90] Penney K.L., Schumacher F.R., Kraft P., Mucci L.A., Sesso H.D., Ma J., Niu Y., Cheong J.K., Hunter D.J., Stampfer M.J. (2011). Association of KLK3 (PSA) Genetic Variants with Prostate Cancer Risk and PSA Levels. Carcinogenesis.

[91] Diesch J., Le Pannérer M.-M., Winkler R., Casquero R., Muhar M., van der Garde M., Maher M., Herráez C.M., Bech-Serra J.J., Fellner M. (2021). Inhibition of CBP Synergizes with the RNA-Dependent Mechanisms of Azacitidine by Limiting Protein Synthesis. Nat. Commun..

[92] Hammitzsch A., Tallant C., Fedorov O., O’Mahony A., Brennan P.E., Hay D.A., Martinez F.O., Al-Mossawi M.H., de Wit J., Vecellio M. (2015). CBP30, a Selective CBP/P300 Bromodomain Inhibitor, Suppresses Human Th17 Responses. Proc. Natl. Acad. Sci. USA.

[93] Hay D.A., Fedorov O., Martin S., Singleton D.C., Tallant C., Wells C., Picaud S., Philpott M., Monteiro O.P., Rogers C.M. (2014). Discovery and Optimization of Small-Molecule Ligands for the CBP/P300 Bromodomains. J. Am. Chem. Soc..

[94] Garcia-Carpizo V., Ruiz-Llorente S., Sarmentero J., Graña-Castro O., Pisano D.G., Barrero M.J. (2018). CREBBP/EP300 Bromodomains Are Critical to Sustain the GATA1/MYC Regulatory Axis in Proliferation. Epigenetics Chromatin.

[95] Spriano F., Gaudio E., Cascione L., Tarantelli C., Melle F., Motta G., Priebe V., Rinaldi A., Golino G., Mensah A.A. (2020). Antitumor Activity of the Dual BET and CBP/EP300 Inhibitor NEO2734. Blood Adv..

[96] Canales T.M., van Gils N., Vermue E., Rutten A., Denkers F., van der Deure T., Giles F., Smit L. (2019). PS967 Preclinical activity of the novel oral dual bet-CBP/P300 inhibitors, NEO1132 and NEO2734, in acute myeloid leukemia. Hemasphere.

[97] van Gils N., Martiañez Canales T., Vermue E., Rutten A., Denkers F., van der Deure T., Ossenkoppele G.J., Giles F., Smit L. (2021). The Novel Oral BET-CBP/P300 Dual Inhibitor NEO2734 Is Highly Effective in Eradicating Acute Myeloid Leukemia Blasts and Stem/Progenitor Cells. Hemasphere.

[98] Filippakopoulos P., Qi J., Picaud S., Shen Y., Smith W.B., Fedorov O., Morse E.M., Keates T., Hickman T.T., Felletar I. (2010). Selective Inhibition of BET Bromodomains. Nature.

[99] Jiang G., Deng W., Liu Y., Wang C. (2020). General Mechanism of JQ1 in Inhibiting Various Types of Cancer. Mol. Med. Rep..

[100] Picaud S., Fedorov O., Thanasopoulou A., Leonards K., Jones K., Meier J., Olzscha H., Monteiro O., Martin S., Philpott M. (2015). Generation of a Selective Small Molecule Inhibitor of the CBP/P300 Bromodomain for Leukemia Therapy. Cancer Res..

[101] Strachowska M., Gronkowska K., Michlewska S., Robaszkiewicz A. (2021). CBP/P300 Bromodomain Inhibitor–I–CBP112 Declines Transcription of the Key ABC Transporters and Sensitizes Cancer Cells to Chemotherapy Drugs. Cancers.

[102] Ogryzko V.V., Schiltz R.L., Russanova V., Howard B.H., Nakatani Y. (1996). The Transcriptional Coactivators P300 and CBP Are Histone Acetyltransferases. Cell.

[103] Bannister A.J., Kouzarides T. (1996). The CBP Co-Activator Is a Histone Acetyltransferase. Nature.

[104] Puttagunta R., Tedeschi A., Sória M.G., Hervera A., Lindner R., Rathore K.I., Gaub P., Joshi Y., Nguyen T., Schmandke A. (2014). PCAF-Dependent Epigenetic Changes Promote Axonal Regeneration in the Central Nervous System. Nat. Commun..

[105] Yokomizo C., Yamaguchi K., Itoh Y., Nishimura T., Umemura A., Minami M., Yasui K., Mitsuyoshi H., Fujii H., Tochiki N. (2011). High Expression of P300 in HCC Predicts Shortened Overall Survival in Association with Enhanced Epithelial Mesenchymal Transition of HCC Cells. Cancer Lett..

[106] Ebrahimi A., Sevinç K., Gürhan Sevinç G., Cribbs A.P., Philpott M., Uyulur F., Morova T., Dunford J.E., Göklemez S., Arı Ş. (2019). Bromodomain Inhibition of the Coactivators CBP/EP300 Facilitate Cellular Reprogramming. Nat. Chem. Biol..

[107] Petrif F., Giles R.H., Dauwerse H.G., Saris J.J., Hennekam R.C.M., Masuno M., Tommerup N., van Ommen G.-J.B., Goodman R.H., Peters D.J.M. (1995). Rubinstein-Taybi Syndrome Caused by Mutations in the Transcriptional Co-Activator CBP. Nature.

[108] Cheng G., Liu F., Asai T., Lai F., Man N., Xu H., Chen S., Greenblatt S., Hamard P.-J., Ando K. (2017). Loss of P300 Accelerates MDS-Associated Leukemogenesis. Leukemia.

[109] Lasko L.M., Jakob C.G., Edalji R.P., Qiu W., Montgomery D., Digiammarino E.L., Hansen T.M., Risi R.M., Frey R., Manaves V. (2017). Discovery of a Selective Catalytic P300/CBP Inhibitor That Targets Lineage-Specific Tumours. Nature.

[110] Sayapina M.S., Savelov N.A., Karseladze A.I., Bulanov A.A., Tryakin A.A., Nosov D.A., Garin A.M., Tjulandin S.A. (2016). Nuclear Protein of the Testis Midline Carcinoma Masquerading as a Primary Mediastinal Seminoma. Rare Tumors.

[111] Zhang X., Zegar T., Lucas A., Morrison-Smith C., Knox T., French C.A., Knapp S., Müller S., Siveke J.T. (2020). Therapeutic Targeting of P300/CBP HAT Domain for the Treatment of NUT Midline Carcinoma. Oncogene.

[112] Yang Y., Zhang R., Li Z., Mei L., Wan S., Ding H., Chen Z., Xing J., Feng H., Han J. (2020). Discovery of Highly Potent, Selective, and Orally Efficacious P300/CBP Histone Acetyltransferases Inhibitors. J. Med. Chem..

[113] Bowers E.M., Yan G., Mukherjee C., Orry A., Wang L., Holbert M.A., Crump N.T., Hazzalin C.A., Liszczak G., Yuan H. (2010). Virtual Ligand Screening of the P300/CBP Histone Acetyltransferase: Identification of a Selective Small Molecule Inhibitor. Chem. Biol..

[114] Cai L.-Y., Chen S.-J., Xiao S.-H., Sun Q.-J., Ding C.-H., Zheng B.-N., Zhu X.-Y., Liu S.-Q., Yang F., Yang Y.-X. (2021). Targeting P300/CBP Attenuates Hepatocellular Carcinoma Progression through Epigenetic Regulation of Metabolism. Cancer Res..

[115] Ogiwara H., Sasaki M., Mitachi T., Oike T., Higuchi S., Tominaga Y., Kohno T. (2016). Targeting P300 Addiction in CBP-Deficient Cancers Causes Synthetic Lethality by Apoptotic Cell Death Due to Abrogation of MYC Expression. Cancer Discov..

[116] Giotopoulos G., Chan W.-I., Horton S.J., Ruau D., Gallipoli P., Fowler A., Crawley C., Papaemmanuil E., Campbell P.J., Göttgens B. (2016). The Epigenetic Regulators CBP and P300 Facilitate Leukemogenesis and Represent Therapeutic Targets in Acute Myeloid Leukemia. Oncogene.

[117] Huang X., Yan J., Zhang M., Wang Y., Chen Y., Fu X., Wei R., Zheng X., Liu Z., Zhang X. (2018). Targeting Epigenetic Crosstalk as a Therapeutic Strategy for EZH2-Aberrant Solid Tumors. Cell.

[118] Roth S.Y., Denu J.M., Allis C.D. (2001). Histone Acetyltransferases. Annu. Rev. Biochem..

[119] Stimson L., Rowlands M.G., Newbatt Y.M., Smith N.F., Raynaud F.I., Rogers P., Bavetsias V., Gorsuch S., Jarman M., Bannister A. (2005). Isothiazolones as Inhibitors of PCAF and P300 Histone Acetyltransferase Activity. Mol. Cancer Ther..

[120] Skehan P., Storeng R., Scudiero D., Monks A., McMahon J., Vistica D., Warren J.T., Bokesch H., Kenney S., Boyd M.R. (1990). New Colorimetric Cytotoxicity Assay for Anticancer-Drug Screening. JNCI J. Natl. Cancer Inst..

